# Towards Resilient Health Systems in Sub-Saharan Africa: A Systematic Review of the English Language Literature on Health Workforce, Surveillance, and Health Governance Issues for Health Systems Strengthening

**DOI:** 10.5334/aogh.2514

**Published:** 2019-08-16

**Authors:** Martin Amogre Ayanore, Norbert Amuna, Mark Aviisah, Adam Awolu, Daniel Dramani Kipo-Sunyehzi, Victor Mogre, Richard Ofori-Asenso, Jonathan Mawutor Gmanyami, Nuworza Kugbey, Margaret Gyapong

**Affiliations:** 1 School of Public Health, University of Health and Allied Sciences, Ho, GH; 2 Legon Centre for International Affairs and Diplomacy, University of Ghana, Accra, GH; 3 Department of Health Professions Education and Innovative Learning, School of Medicine and Health Sciences, University for Development Studies, Tamale, GH; 4 Monash University, Department of Epidemiology and Preventive Medicine, Melbourne, AU; 5 Institute of Health Research and Professor, University of Health and Allied Sciences, Ho, GH

## Abstract

**Background:**

Meeting health security capacity in sub-Saharan Africa will require strengthening existing health systems to prevent, detect, and respond to any threats to health. The purpose of this review was to examine the literature on health workforce, surveillance, and health governance issues for health systems strengthening.

**Methods:**

We searched PubMed, Science Direct, Cochrane library, CINAHL, Web of Science, EMBASE, EBSCO, Google scholar, and the WHO depository library databases for English-language publications between January 2007 and February 2017. Electronic searches for selected articles were supplemented by manual reference screening. The review followed the Preferred Reporting Items for Systematic reviews and Meta-Analyses (PRISMA) guidelines.

**Results:**

Out of 1,548 citations retrieved from the electronic searches, 31 articles were included in the review. Any country health system that trains a cadre of health professionals on the job, reduces health workforce attrition levels, and builds local capacity for health care workers to apply innovative mHealth technologies improves health sector performance. Building novel surveillance systems can improve clinical care and improve health system preparedness for health threats. Effective governance processes build strong partnerships for health and create accountability mechanisms for responding to health emergencies.

**Conclusions:**

Overall, policy shifts in African countries’ health systems that prioritize training a cadre of willing and able workforce, invest in robust and cost-effective surveillance capacity, and create financial accountability and good governance are vital in health strengthening efforts.

## Introduction

In the wake of any health threat, the strength of the health system at the national and international levels is often tested [1,2]. The World Health Organization (WHO) defines health system as “all organizations, people and actions whose primary intent is to promote, restore, or maintain health [3].” As put forward by Heymann et al. [4] there is a need to pay attention to health system challenges at all levels of health care delivery in the context of sub-Saharan Africa (SSA). Many debates and disagreements surround the precise definition of health security despite the universal acceptance of health security as an important public health issue that requires close attention [5]. Health security at the national and the global level is said to exist if the following conditions are met: there is protection against any health threat, there are new adaptations and approaches to new health conditions that may arise, new actors are engaged, including military establishments, and synergy is drawn between foreign policy and public health at the national and global levels [5]. Global public health security aims to minimize vulnerability to public health threats across geographic regions and international boundaries [6]. To attain global health security, attaining individual health security at any country level is vital [7]. A country’s health system that is made resilient through strengthening core building blocks in the health care system can promote the capacity of global health security [3].

In SSA, there is renewed interest in strengthening national health systems to make them resilient to meet national and global health threats [1,2,4]. Health system strengthening refers to a change in health performance with the goal of attaining efficiency and effectiveness in the health system [8]. Kruk et al. defined health system resilience as the capacity of health actors, institutions, and populations to effectively prepare for and respond to the public health consequence of any health threat, with the aim of protecting human life and ensuring good health outcomes during and after crises [1]. In 2014, the Ebola outbreak in West Africa was reported as a failure of leadership at many levels [9]. Some identified gaps, such as the absence of surveillance and health reporting systems [2,10], weak health systems not adaptable to changing health conditions [11,12], and ineffective health systems at the primary care level [13,14]. In addition, the absence of vital registration systems [15,16] and clinic-oriented health system designs which are slow in responding to new epidemiologic and disease threats [17,18] were also reported as gaps in the health system in areas where many died during the Ebola crises. The functionality of health systems have improved in some SSA contexts [19,20,21], while other settings have witnessed slow health system improvements [22,23] amidst several health challenges [21,24]. Fundamentally, having an adequate health workforce, health surveillance, and health leadership and governance are identified as major factors in strengthening health systems in SSA [3], in addition to other health system building blocks [25] required to make health systems more resilient now and in the future [26].

This systematic review examined the existing English language literature on health workforce, surveillance, and governance capacity for strengthening health systems in the SSA region. The review addressed the question: how can health systems in SSA be resilient, with regards to the role of the health workforce, surveillance systems, and governance play in the strengthening of health systems? This review is based on the premise that adequate and skilled health workforce, surveillance systems, and governance capacity are vital to attaining resilient health systems [7,27]. Evidence from this review will add to exiting literature on strengthening health systems in SSA. The review is structured as follows: a detailed methodology outlines the search process, including inclusion and exclusion criteria, quality appraisals, and data extraction, followed by synthesis and discussion of studies reviewed.

## Methods

### Study design and framework for review

We conducted a systematic literature review following guidelines published by *Lancet* [28], and the Preferred Reporting Items for Systematic reviews and Meta-Analyses [29] (PRISMA).

### Search strategy and selection criteria

**Figure 1:**
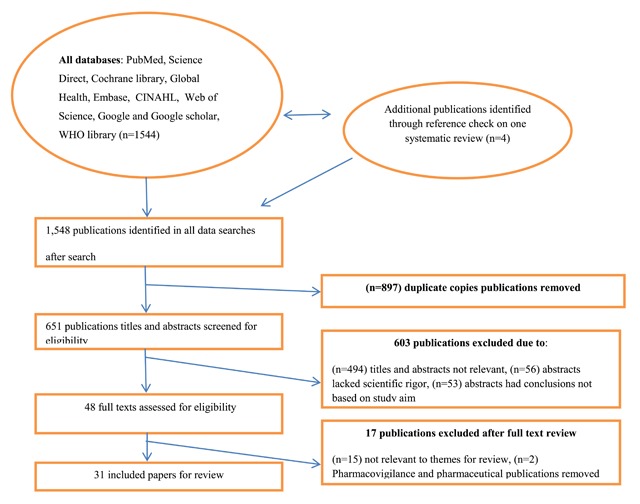
PRISMA flow diagram showing how publications were screened for eligibility for inclusion.

Our search strategy was informed by three out of the six core elements of the WHO health system building blocks. These are the health workforce, health information systems, and leadership and governance [3]. The following keywords were used in the search: health workforce, surveillance system, health governance, health system strengthening, resilient health system, and sub-Saharan Africa. All possible synonyms were generated for the main key words and included in the search using Medical Subject Headings (MsSH) in PubMed. The search was conducted in PubMed, Science Direct, Cochrane library, CINAHL, Web of Science, EMBASE, and EBSCO for literature published between January 2007 and February 2017. Google scholar and the WHO library (WHOLIS) were searched for country-level policy documents and protocols on health system strengthening using the same key words above. Figure 1 shows the PRISMA flow diagram of the publications screened prior to the final selection of reviewed publications. All identified articles were first screened by two authors (MAA, MA) for titles and abstracts, and full-text assessment was carried out for eligibility and inclusion. The reference lists of publications were also searched to identify eligible publications for inclusion.

### Inclusion and exclusion criteria

English-language literature that presented findings on health workforce, surveillance, and governance capacities for strengthening health systems in SSA were included. We included health system capacity and gaps identified during the Ebola crises in West Africa. Although the search strategy covered publications across SSA whose populations speak different languages, only English language publications were included in the review.

To avoid data that may not fit our aim, we excluded publications on disease-specific conditions that have not been documented as an epidemic in SSA. Publications on food security and health, climate change, and bioterrorism interlinkages as other broader determinants of health security and a measure of resilience to the health system were excluded. Clinical or trial studies on health system strengthening were excluded. Publications related to pharmacovigilance were excluded. Non-English literature was excluded in the review because the research team were all English native speakers.

### Screening and extraction

To minimise bias in our data extraction process, two authors (MAA, NA) independently screened the initial publications for eligibility and inclusion for review. The two authors independently identified six publications that were difficult to classify into the three areas under the review because they presented cross-cutting issues reflecting in all three areas of interest. Four out of six publications were resolved through consensus-based discussions. A third author (VM) assisted in resolving classification disagreements of two publications among MAA and NA. From each eligible publication, the following information was extracted: publication origin and year, sample demographic, study title, design and setting, analytical approach, and main findings on health workforce, surveillance, and governance issues for health system strengthening for SSA.

### Data synthesis

Narrative synthesis approach was used to summarize the findings. This procedure was preferred because it applies a textual approach to data synthesis and story-telling [30]. Five team members (MA, AA, NK, DDK, and JG) independently coded tabulated findings on the three areas of the review and mapped out common codes, concepts, and categories. Two other team members (VM, RO) built upon five team members’ work by categorizing and synthesizing salient and emergent codes into final relevant themes. Two other team members (MAA, JG) cross validated all synthesized findings and resolved any discrepancies, ensuring findings reflected the three areas under review.

### Study quality appraisal

We appraised the internal validity of individual articles using the Crowe Critical Appraisal Tool (CCAT). This technique for checking quality was chosen because of its usefulness for diverse research designs [31]. Applying eight domain elements under CCAT, and following the procedure conducted by Crowe et al. [32] we appraised and scored each domain element within a range of 0–5. The average score for all 31 studies was 4, indicating a high quality CCAT across all studies.

## Results

### General characteristics of studies

A total of 1,548 publications were retrieved across all databases. In applying our inclusion and exclusion criterion, 31 English-language publications in SSA countries qualified for full review, as presented in Figure 1. Table 1 shows the list of publications and the African regions included in the review. A detailed breakdown is provided as a supplementary file. Three authors (MA, GJ, VM) screened and classified publications into the three areas guided by the WHO framework on health system strengthening that fell within the scope of this review. Three publications were classified under health workforce, 10 publications under surveillance capacity, and 18 publications under governance.

**Table 1:** List and origins of publications reviewed.

SSA Region	Number of publications

East Africa	9
West Africa	10
Southern Africa	6
Global perspective with focus on SSA	6
Total	31

### Health workforce capacity for health system strengthening

Findings on health workforce capacity for health system strengthening are presented in Table 2. Three publications were reviewed regarding health workforce. The studies were based in Rwanda, Nigeria, and Botswana. The three publications [33,34,35] focused on health workforce for health system strengthening in the country context. Four critical issues were drawn from these publications: (1) continual job training for the health workforce yields better results compared to long term offsite training of a cadre of the health workforce [34], (2) planning for attrition should be anticipated within African health systems, with rigorous development of strategies for attrition, recruitment, and training [33,34,35] (3) mHealth technologies can improve community health workers’ (CHW) health service delivery capacity in low resource settings [35], and (4) Improving managerial skills and competencies of cadre of workforce to effectively generate and use reliable data to inform health system improvement needs at country level [34].

**Table 2:** Health workforce-related publications included in review.

Reference Publication	Study Title	Design/Setting/Data/Analytical Approach	Main findings on local health system strategies

Ledikwe et al. 2013 [34], Botswana	Establishing a health information workforce: innovation for low- and middle-income countries	Mixed method approach with qualitative and quantitative data was used. Tools included pre and post-test, interviews during stakeholder site visits, a survey focusing on achievements, focus group discussions, and an attrition assessment	- Prompt on the job training yields better results and response on health information management and needs compared to long term offsite training for health information personnel. - Planning for attrition through development of strategies for efficient recruitment and development of training materials that could easily be used to train new staff is important to enhance workforce numbers and improvements for health systems.
Otu et al. 2016 [35], Nigeria	Using a mHealth tutorial application to change knowledge and attitude of frontline health workers to Ebola virus disease in Nigeria: A before-and-after study	Quantitative cross-sectional survey in 14 health facilities in Ondo state, Nigeria	- mHealth tutorial applications from this study show modest changes in knowledge and attitudes of health care workers post project implementation. - mHealth technologies could be effectively used to disseminate information and train community health workers working in remote and far to reach country settings.
Sayinzoga et al. 2016 [33], Rwanda	Drivers of improved health sector performance in Rwanda: A qualitative view from within	Web-based survey among district health managers on opinions that drive performance in the health sector	- Community health workers and health insurance come out as factors that are considered to have contributed most to Rwanda’s remarkable achievements in the past decade. - Managerial skills and capacities of health staff and the culture of continuous monitoring of key indicators by an active workforce is critical for good progress on health outcomes.

A study in Botswana found continual job trainings on health information systems (HIS) used for tracking and reporting health indices was effective in improving patient care outcomes [34]. The absence of clear administrative roles and command structures can lead to high health workforce attrition rates, particularly in health emergencies. Clearer roles, responsibilities, job security, and appropriate career trajectories for both old and new cadres of health professionals can reduce attrition rates in the health sector. One study [35] reported on-the-job trainings for CHWs can improve health worker responsiveness to dealing with emergency health threats in Botswana. Additionally, effective monitoring of health worker support roles in the health service delivery system can bring good health returns, as reported in Rwanda [33]. Overall, the three publications highlighted the need for country health systems to address technical and operational concerns in recruitment, training, and retention of a health workforce willing and able to support, prevent, detect, and respond to any health threats within the health care delivery system.

### Surveillance and health information capacity for health systems strengthening

Table 3 summarizes the findings on surveillance capacity for health system strengthening. In Kenya and Uganda, strong surveillance investments improved the strength of the health system [36]. Two publications [37,38], one on malaria surveillance in endemic regions in SSA and the other on the Guinean Ebola crises, reported the importance of remote sensing and early warning systems for improving future health emergencies in Africa. The application of appropriate and sustainable case monitoring systems acts as a means of both providing reliable information and building data for the health systems, as evident during the Ebola crises in Guinea [38].

**Table 3:** Surveillance and data management-related publications reviewed.

Reference Publication	Study Title	Design/Setting/data/Analytical Approach	Main findings on local health system strategies

Martha Gyansa-Lutterodt 2013 [39], Ghana	Antibiotic resistance in Ghana	Comment on antibiotic use and its growing resistance in Ghana	- Low capacity of linking laboratory diagnostic tests to selection of medicines for treatments exist. - Uncontrolled antibiotic use for agriculture and veterinary purposes is growing and likely to increase drug use resistance. - An improved surveillance on drug supply and use and regulatory mechanisms for improving antibiotic use will go a long way to improve most health systems in Africa.
Justine Davis et al. 2017 [40], Africa	Sustainable clinical laboratory capacity for health in Africa	Comment on laboratory capacities across Africa	- Targeting and training a cadre of multi-skilled health professionals to work in laboratories that can deal with broad range of health conditions (communicable and non-communicable) are crucial for promoting individualized health security needs.
Lancet Editorial 2017 [41], Africa	Global health security: How can laboratories help?	Editorial comment with focus in Africa	- There is need to actively promote and support laboratories to be reliable both in diagnostic and treatments. Providing laboratory leadership seminars and training programmes is important to improve and guarantee this objective.
Jones et al. 2008 [36], Kenya and Uganda	District-based malaria epidemic early warning systems in East Africa: Perceptions of acceptability and usefulness among key staff at health facility, district and central levels	Development and testing of a district-based malaria surveillance system in four pilot districts of Kenya and Uganda. Health staff interviews conducted among 52 health staff at districts and Ministries of Health in Kenya and Uganda	- The system transfer of responsibility to district level manpower resulted in perceptions of empowerment among district-based health staff. - Improved support together with transfer of responsibility helped to sustain motivation and improved surveillance on malaria tracking and control. - Increased logistical support is vital in the midst of increased participation and involvement to sustain surveillance gains.
Cox et al. 2007 [37], Africa	Early warning systems for malaria in Africa: From blueprint to practice	Review of evidence in Africa	- The development of appropriate and sustainable case monitoring systems can act both as means to providing reliable information and building data for the health systems vital in poor settings with poor data or non-existent malaria data systems.
Peckham et al. 2017 [38], West Africa	Satellite and the new war on Infection: Tracking Ebola in West Africa	Data synthesis of available evidence of study aim	- Remote sensing could be applied to track disease and monitor isolated rural communities, providing mapping data that supports on-the-ground logistics and contract tracing as evidenced during the Ebola crises.
Haskew et al. 2015 [42], Kenya	Implementation of a Cloud-Based Electronic Medical Record to Reduce Gaps in the HIV Treatment Continuum in Rural Kenya	Project evaluation of an electronic medical record systems for HIV cases in rural Kenya	- Cloud based electronic medical system provides for real-time access to anonymised data beyond the level of the clinic to inform timely decision making on HIV interventions. - The system proved to be cost effective, scalable compared to other local context models.
Kiberu et al. 2014 [43], Uganda.	Strengthening district-based health reporting through the district health management information software system: The Ugandan experience	Training facilitation for cadre of health professional on the use of the district health management information software system version 2 (DHIS2) across 112 districts.	- Training resulted in timeliness and completeness in health reporting of routine outpatient, inpatient, and health service usage data from district to the national. - Onsite support and training for data managers and professional in addition to removing logistical constraints improves data management efficiency at district level.
Mate et al. 2009 [44], South Africa	Challenges for Routine Health System Data Management in a Large Public Programme to Prevent Mother-to-Child HIV Transmission in South Africa	A survey conducted between January-December 2007 on completeness and accuracy of HIV data for decision making in South Africa	- System improvements must improve front line health staff skills/knowledge on routine quality data, adopt use of simplified data tools, maintain minimum set of indicators and registers.
Wong et al. 2009 [45], Ethiopia	Developing patient registration and medical records management system in Ethiopia	Pre-post intervention study in large referral hospital	- The evidence showed merging of patient registrations and medical records into one process, designing master patient index and improved filing procedures together with adequate training of human resources are vital to guarantee improved patient care services.

Weak epidemiologic data gathering and application was reported in three publications [39,40,41] as a threat to combating future health threats at country levels. Investments in novel surveillance systems, such as the cloud-based electronic medical systems, was reported in Kenya [42] to have improved clinical outcomes among HIV patients. Three publications in Uganda, South Africa, and Ethiopia [43,44,45] reported that innovative data management systems used at the lower level of the health system structure can improve primary health care outcomes. Novel data managing processes, such as merging patient registrations and medical records into a unified process, assists in providing a timely response for patient care outcomes in Ethiopia [45].

Reliable data generation and use can support monitoring and controlling growing disease resistance for opportunistic infections such as HIV and tuberculosis [46]. Incompleteness and poor data accuracy poses a challenge for improving HIV patient outcomes, as reported in South Africa [47]. In Ethiopia, a study reported prompt, efficient, and timely medical record documentation was vital to improving population health outcomes [45]. Onsite support and training for data managers and professionals can improve data management efficiency [43]. Additionally, electronic record management systems can aid in generating alerts and reports on health service outcomes [37,43] and strengthen health system preparedness to handle health threats [45,47]. In addition, the application of problem solving and quality improvement techniques can improve population health outcomes [43,45,47].

### Governance capacity for health systems strengthening

One publication in the West Africa region [4], shown in Table 4, reported health resource availability and allocation is both a technical and political problem. Health systems with effective communication and support channels can support building leadership capacities in the management of health services [48]. Also, the development of a national-level transformative initiative for health risk mitigation is vital for health system improvements [49]. Three publications [3,10,25] reported the need for building strong partnerships and improving stewardship and management of resources to deliver good health outcomes at a population level. Criterion-based audits as a public sector performance management tool can improve health deliverables, as evident in Malawi [50]. A study in Zambia reported community dialoguing and the application of provider and patient score cards were cost-effective approaches for accountability in the health sector [51].

**Table 4:** Health Governance-related publications included in review.

Reference Publication	Study Title	Design/Setting/Data/Analytical Approach	Main findings on local health system strategies

Heymann et al. 2016 [4], Lancet, West Africa	Global Health security: Wider lessons from the West African Ebola Virus disease epidemic	Historical and secondary review of Ebola events in West Africa during and post Ebola	- Health systems must pay attention to individual health security needs. Resource availability and allocation is both a technical and political problem for most African countries. - Adequate funding for research and development and access to safe vaccines are among pivotal vehicles to driving an effective health system. - Poor political commitments at governments level is worrying and not in the best interest of a growing African population.
Drobac et al. 2013 [76], Rwanda	Comprehensive and integrated district health systems strengthening: The Rwanda population Health Implementation and Training (PHIT) Partnership	Impact evaluation using population level outcome data from demographic health surveys (DHS) in Rwanda (protocol)	- Mentorship and enhanced supervision (MESH) of health staff improves quality care at health facilities. - The use of community health household registers to track activities and improve reporting at facility levels improves health system outcomes. - The use of electronic record management systems for generating alerts and reporting improves quality of care at facility centres.
Sherr et al. 2013 [48], Mozambique	Strengthening integrated primary health care in Sofala, Mozambique	Evaluation design technique employing a quasi-experimental controlled time-series design to assess impact of partnership strategy on under-5 mortality rates in study setting	- In health systems with multiple district level partners, building communication and support channels across all levels of the health system with partners is vital to success. - Applying practical skills-based training approaches in management serves as useful and feasible technique to build leadership capacity across multiple districts/regions in a country.
Cho et al. 2014 [85], Global Editorial	Out of Africa, Into Global Health Security Agenda	Editorial comment	- Investments for the development of suitable and effective vaccines is key for preparedness in any health emergencies. - Patient safety issues are important to the efficacy of preventing potential accidents during disease outbreaks in Africa.
Cho et al. 2015 [86], Global Editorial	Two Epidemics and Global Health Security Agenda	Editorial comment	- Holistic country and continental assessment of health system gaps along multiple areas is needed to improve long term health security needs. - Ebola and MERS showed health is directly related to national security issues, emphasizing commitment at all levels for ensuring early detection, prevention, and rapid response to biological threats to human health.
GHSA Task Force Team 2015 [87], Brief Report	Global Health Security: The Lessons from the West African Ebola Virus Disease Epidemic and MERS outbreak in the Republic of Korea	Retrospective assessment of Ebola and MERS	- Effective health systems are those in use every day and capable of scaling up in emergencies. - Despite nascent capacity in Nigeria, a well-coordinated surveillance and laboratories emergency operations by trained health staff enabled the control of Ebola.
Patel et al. 2015 [49], West Africa	Health security and political and economic determinants of Ebola	Correspondence on global health security and its needs	- Country and global political and economic determinants to health can be tackled multi-sectoral and may not involve only one government(s) level intervention. - National level consensus on transformative initiatives with potential to mitigate risk are necessary to curtail any future health emergencies.
Wang et al. 2013 [52], Global Review	New vaccine introductions: Assessing the impact and the opportunities for immunization and health systems strengthening	Mixed approaches that includes a review of published and grey literature, in-depth case studies in three countries, interviews with key informants from countries and WHO Regional Offices, and a multivariable analysis examining impact of NVI on coverage for 3rd dose of diphtheria–tetanus–pertussis vaccine (DTP3).	- High-quality monitoring and evaluation, including disease - surveillance and immunization coverage monitoring, resource, performance, and management accountability. - Viewing health service delivery in a holistic and integrated manner rather than as independent, disparate programmes is recognized as necessary in order to achieve efficiencies and avoid fragmentation. - A strong, country-led, evidence-based decision-making, - planning, and prioritization process that is accountable and - coordinated with other components of the health system.
Buseh et al. 2015 [53], West Africa	The Ebola Epidemic in West Africa: Challenges, opportunities and policy priority areas	Literature review of peer-reviewed journals on disease burden and health reforms in developing countries with focus on West Africa	- Promoting family-centered approaches in emergencies could help control infection and re-infection rates during disease outbreaks such as Ebola in West Africa. - Public-private partnerships to deliver reliable and efficient basic health services, in addition to the development of affordable vaccines and drugs. - Identifying and strengthening formal and informal African institutions will improve the resilience of countries to respond in health emergencies.
Gostin et al. 2015 [88], Lancet West Africa	A retrospective and prospective analysis of the West African Ebola virus disease epidemic: Robust national health systems at the foundation and an empowered WHO at the apex	A synthesis of public policy issues in post-Ebola outbreak in West Africa	- An empowered WHO at the apex, with well-coordinated funding and actions among many parties, is important to improve health emergences and vulnerabilities, particularly in poor resourced settings.
Siekmans et al. 2017 [89], Liberia	Community-based health care is an essential component of a resilient health system: Evidence from Ebola outbreak in Liberia	A descriptive observational study design using mixed methods to collect data among community health workers.	- Community health workers knowledge of cultural and social complexities at community centres enhances patients trust and confidence in the health system during emergencies. - In critical emergencies, adequate training and guidelines with supportive supervision to community health workers help deliver lifesaving services to patients.
Kongnyuy et al. 2008 [50], Malawi	Criteria-based audit to improve a district referral system in Malawi: A Pilot study	A criteria-based audit by conducting a retrospective review of 60 obstetric emergencies from 12 health centres	- Criterion-based audit is scalable in poor resource settings where referral care is vital for obstetric care. - Local health actors support, and strong involvement is vital for, sustaining health system gains at long term.
Topp et al. 2015 [51], Zambia	The impact of human immunodeficiency virus (HIV) service scale-up on mechanisms of accountability in Zambian primary health centres: A case-based health systems analysis	Guided by the Mechanisms of Effect framework and Brinkerhoff’s work on accountability. In-depth multi-case study with case data interviews collected among providers. Direct observation and key informant interviews were also used	- Resource-intensive investments in HIV services contributes to improving administrative answerability and improving capacities to deliver and monitor service delivery. - Community dialoguing, patient score cards are local level approaches used to improve micro-level (people-centeredness) for services delivery.
Coovadia et al. 2009 [54], South Africa	The health and health system of South Africa: Historical roots of current public health challenges	Historical review of South African Health Care Systems	- Programmes with direct impact on the social determinants of health and development (stigma, subordination of women, poverty and inequality, violence, and traditional practices) have positive effects on health systems functioning. - Macroeconomic policies that promote growth alone are insufficient for health systems effectiveness. An economic architecture that allows the development of programmes that reduce poverty, unemployment, and inequities are essential for health systems strengthening.
Kieny et al. 2014 [10], Western Africa	Health-system resilience: Reflections on the Ebola crisis in western Africa	Perspectives on the health systems in affected West African countries	- The Ebola outbreak must trigger substantial investments and adequate reforms in the worst-affected countries health systems. National governments, assisted by external partners, need to develop and implement strategies to make their health systems stronger and more resilient. - In the short-term, nongovernmental organizations, civil society, and international organizations will have to bolster the national health systems, both to mitigate the direct consequences of the outbreak and to ensure that all essential health services are being delivered.
WHO 2010 [25]	Monitoring the building blocks of health systems: A handbook of indicators and their measurement strategies	A published book on the essential building blocks in health systems strengthening	- Summary points: Strong and effective health systems at country levels are the foundations to ensuring global health security. Appropriately applying standardized measures and indicators to appraise health systems within the global context will support in planning and health prioritization in national health system needs.
WHO 2007 [3]	Everybody’s business: Strengthening health systems to improve health outcomes: WHOs framework for action	A published book on making health systems strengthening a priority for all citizens, academics, WHO staff, governments, and donors.	- Summary points: There is need for pragmatic synergy among various technical working relationships in WHO. In developing strategies for country health systems, synergy strategies should be pursued in care provision, monitoring of indicators and outcomes, health financing and improving quality of care for patients and the health workforce.

Topp and colleagues in Zambia [51] reported the need for health systems to create administrative answerability mechanisms to monitor and deliver health services, particularly during emergencies. Delivering health services in a holistic and integrated manner can help avoid resource fragmentation and improve efficiency in health care delivery [48,51,52]. Furthermore, public-private partnerships (PPP) are required to address unmet health service demands in Africa [48,53]. Policies that address broader determinants of health, such as poverty, inequality, and violence, are vital to enable health systems to function effectively [54].

Identifying and prioritizing cost-effective interventions during emergencies can control infection and re-infection rates during disease outbreaks [53]. At the apex of global health governance, strong partnership with the WHO and national and global actors is required to avoid late responsiveness, such as during the Ebola crises [55]. Timely and prompt global response at national and international levels by multiple actors is important in pointing the way to meeting national and global health systems goals.

## Discussion

This review covered only eligible English-language literature that examined health workforce, surveillance, and governance capacities for strengthening health systems in SSA. This section discusses country findings in SSA and compares them to those in other countries outside the scope of this review that share similar country level characteristics.

### Health workforce capacity for strengthening health systems

Low technical and operational human resource capacity for health is not a new phenomenon and has been reported in other SSA studies as vital for strengthening health systems [13,56]. Continual on-the-job training for health professionals is a better option compared with long term offsite training of the health workforce, particularly during emergencies. Our results suggest the need for a cadre of health professionals who are adequately trained in the concepts of monitoring and evaluation, in addition to clinical skills, to strengthen country level health systems.

A well-trained monitoring and evaluation workforce is also critical for providing a culture of continuous assessment of key health indicators at points of service delivery [33]. Health information officers’ ability to acquire and apply knowledge on cost-effective monitoring and evaluation methodologies can enhance frontline health workers’ abilities to collect, analyse, and interpret data for quality reporting on health indices [34,57,58,59,60]. This review has identified the need for health systems to generate reliable and timely data on health workforce gaps useful in making timely decisions regarding human resource capacities requirements to address unmet health service demands in SSA.

The review found mHealth technologies to be effective in some low-resource settings for disseminating information and meeting training capacity of community health workers through (1) improving knowledge and attitudes on clinical care and (2) enabling the delivery of essential medicines and information to distant population groups, especially during emergencies. Growing evidence corroborates mHealth’s role for strengthening health systems in Africa [61,62,63,64]. The WHO indicates that low knowledge and information on mHealth applications is a challenge in Africa, hence the need for investments to assess its use and scale-up [65].

Fundamentally, no single strategy can address all the health workforce challenges Africa faces today; a mix of strategies and interventions are required. Task shifting, performance-based payments, and results-based financing as strategies for improving skilled health professional numbers have been sparsely piloted in Rwanda, the Democratic Republic of Congo, South Africa, and Kenya [66,67,68,69,70]. Different models for attracting, recruiting, training, and retaining a critical health workforce must be experimented at country levels.

### Surveillance and health information needs for strengthening health systems

This review highlighted the need for investments that will build robust epidemiologic systems to anticipate future threats in the health system [46,71]. The findings from this review point to the need to initiate or strengthen PPP arrangements that can support building country level capacities and laboratory infrastructure to improve disease surveillance, diagnosis, and treatments.

Health systems that invest in developing surveillance systems for data gathering, monitoring, and feedback can improve disease tracking and control in SSA [72,73]. Studies from developing countries also support the positive effects of satellite imaging for health service delivery [74,75]. Furthermore, linking facility level health data to broader determinants of health at the population level can support tracking any potential health threats before they become epidemics [76].

In SSA, the adoption and use of electronic medical techniques can provide timely detection and response to any health threats to the health system. Our review found that novel electronic and data processing techniques helped to improve HIV treatment outcomes in Kenya [42], as well to improve disease infection control and prevention at the health facility level. This evidence is corroborated by a study in Guinea, Liberia, and Sierra Leone [77]. Their use and advancement to improve data accuracy, as the case reviewed from South Africa, is also important for most African health systems.

Novel surveillance systems can help solve health problems by introducing data quality improvement techniques at health facility levels to improve patient health outcomes. The evidence from the review points to the need for health managers to invest in designing and monitoring health performance using cost-effective surveillance systems. Novel surveillance systems can facilitate a country’s quick response during a disease emergency, as well as support the design and preparation of health emergency preparedness plans. Evidence from the WHO corroborates this and emphasizes the need for integrated surveillance systems in SSA during health emergencies, as evidenced during the Ebola crises [78].

### Governance capacity for health system strengthening

Two health governance shifts were reported as vital to strengthen health systems in Africa: the political and technical commitments to strengthen health capacities. A key political decision in strengthening health systems is financial commitment at the national level. Despite the Abuja declaration on health financing requirements for African governments, many health systems operate with minimal support from their governments [79]. To deliver real-time health responses to health threats, country level commitments across SSA that will mobilize and allocate resources appropriately to strengthen existing weak health systems are vital. Governments in SSA need to create health systems that are inclusive and provide accountability at all levels of the health structure. To enhance weak accountability systems, administrative answerability mechanisms must be effectively developed. Effective accountability systems can help deliver positive health system outcomes [11,12].

Strengthening health partnerships at country level and across SSA has the potential to address technical and logistical capacity regarding the delivery of health services. Review findings point to the fact local partnership building improves PPP initiatives to support health systems so they function effectively. Strong health partnerships assist to address technical and logistics supply needs and to provide mentorship for country level health system improvements, as reported in a Rwandan publication [76]. Furthermore, health partnerships along multiple district levels can help provide effective health leadership, communication, and support channels across many health sectors [80,81].

Health leadership and governance policies that address broad social determinants needs can yield better health outcomes. Financial investments that address broader determinants of health is a good investment to guarantee global health security [82]. The evidence reviewed from the South African health systems after the Apartheid show strengthening social determinants on HIV stigma, subordination of women, poverty, inequality, reducing violence, and traditional practices can improve health care system capacities and improve quality of life [54]. An inclusive approach should be taken among governments, civil society, and local level actors to advance partnership to drive the delivery of health services.

While a substantial amount of financial commitment at country and global levels are vital to deliver large scale changes to population health systems, other cost-effective strategies exist that can equally deliver timely and responsive health care outcomes for populations. This review found community dialoguing and the application of patient score cards were cost-effective strategies that can improve health service delivery at the micro-level. Applying cost-effective health approaches can help overcome health system constraints [83] and help deliver appropriate health solutions [84] for country health system improvements.

The review has limitations. Although we searched several databases, there is the potential that we might have missed other relevant publications. We acknowledge that our exclusion of other periodicals, protocols, and publications from health development partners may have limited our scope of evidence. Our inclusion criteria for only publications in English from SSA might limit generalisability of our findings across French and Portuguese-speaking countries. Also, we did not find and include any policy document at country levels on health security. We acknowledge this limitation on country level evidence on what strategies exist on improving their health systems. We found most evidence on country examples in West Africa stemming from the Ebola crises. None was examined in North Africa. Other cases of country evidence presented came from East and South African countries. Despite these country or regional coverages, our findings remain relevant and applicable across other countries in SSA that share similar health care system capacity needs or barriers.

## Conclusion

This review re-emphasizes the role of an effective cadre of health care workers, good investments in surveillance for decision making, and strong governance in health as critical for the success of any country health system. A health system that trains a cadre of health professionals on the job, reduces health workforce attrition levels, and builds local capacity for health care workers to apply innovative mHealth technologies in delivering services can improve health worker motivation and support for the health sector. Building novel surveillance systems can improve clinical care and health system preparedness for health threats. A strong health leadership and partnership can help deliver financial and logistical capacity for the effective delivery of essential health services. A mix of strategies in addition to the main areas covered under this review, as advocated by the WHO, is needed for strengthening health system capacities [25]. Overall, policy shifts in African countries’ health systems that prioritize training a cadre of health care workers willing and able to provide timely responses to any disease or health threat, investing in building a robust and cost-effective surveillance capacity, and creating financial accountability in health financing and governance can assist in strengthening country level health capacities to deliver better health outcomes.
